# Effect of 17α-methyltestosterone (MT) on oxidation stress in the liver of juvenile GIFT tilapia, *Oreochromis niloticus*

**DOI:** 10.1186/s40064-016-1946-6

**Published:** 2016-03-15

**Authors:** Yao Zheng, Jianhong Qu, Liping Qiu, Limin Fan, Shunlong Meng, Chao Song, Xuwen Bing, Jiazhang Chen

**Affiliations:** Freshwater Fisheries Research Center, Chinese Academy of Fishery Sciences, No. 9 Shanshui East Rd., Wuxi, 214081 Jiangsu China; Scientific Observing and Experimental Station of Fishery Resources and Environment in the Lower Reaches of the Changjiang River, Wuxi, 214081 China; Wuxi Fisheries College, Nanjing Agricultural University, Wuxi, 214081 China

**Keywords:** 17α-Methyltestosterone, Antioxidant enzyme, Genotoxicity, *Oreochromis niloticus*, Oxidative stress

## Abstract

The normal dose of 17α-methyltestosterone (MT) used in fish farming was 60 mg/L, and now the analysis of residual androgens was carried out in waste water obtained from the Beijing area, which could be detected in levels ranging from 4.1 to 7.0 ng/L. For the purpose of aquatic early warning, the present study clearly demonstrated that chronic exposure by higher concentration of MT than environmental relevant concentrations could trigger oxidative stress response to juvenile tilapia by modulating hepatic antioxidant enzyme activities and gene transcription. Some antioxidative parameters (T-GSH, GSH/GSSG and MDA) were significant decreased under 0.5 mg/L MT exposure at 7 and 14 days. Some antioxidant enzymes (SOD, CAT and GST) and transcriptional changes (*sod* and *cat*) were revealed significant decreases for MT treated groups at 7 days. Total antioxidant capacity was significant increased only in 5 mg/L MT exposure groups, but GR activities were not affected all through the whole exposure period. Almost all of the antioxidant enzymatic genes detected in the present study were showed significant increments for MT exposure both at 14 and 21 days, and the genotoxicity profile of antioxidant enzymatic genes were revealed dose-dependent manner. This study presented evidence that MT could result in oxidative stress response in the early stages of GIFT tilapia.

**Electronic supplementary material:**

The online version of this article (doi:10.1186/s40064-016-1946-6) contains supplementary material, which is available to authorized users.

## Background

17α-Methyltestosterone (MT), an artificial androgenic compound, is often used to induce masculinization of both secondary sex characteristics and gonads in aquatic filed studies (Homklin et al. 2009; Golan and Levavi-Sivan 2014; Shen et al. 2015). For example, male tilapia and Yellow catfish has grown faster than females, and MT-immersion for sexually immature hatched larvae has been used to produce mono-male groups. MT also induced organic impairment (Seki et al. 2004) associated with detoxification and antioxidant defense systems in the form of whole cytochrome P450 (CYP) biotransformation (Kim et al. 2014). Some artificial compounds targeted oxidative defense system via hormone biosynthesis and catalytic mechanism of CYP (Mimeault et al. 2006; Ibrahim and Harabawy 2014), and androgenic compounds directly induced antioxidant enzymatic activities and genotoxicity (Larsson et al. 2002).

Oxidative stress is an important manifestation in fish, and the oxidative damage induced by chemicals in aquatic ecosystems can be assessed through the measurements of antioxidant enzyme activities. Androgenic analogues used as oxidative stress inducers to produce excess reactive oxygen species (ROS), which resulted in hepatotoxicity. The main ROS are generated in the mitochondria (Zorov et al. 2006) and ROS detoxification could be provoked both directly by antioxidant enzymes, such as the radical-scavenging enzymes: superoxide dismutase (SOD; EC 1.15.1.1), catalase (CAT; EC 1.11.1.6), glutathione peroxidase (GPx; EC 1.11.1.9), and indirectly by stabilizing the levels of glutathione (GSH) collaboration with the assistance of glutathione-S-transferase (GST; EC 2.5.1.18) and glutathione reductase (GR; EC 1.8.1.7). ROS-induced oxidative stress has been considered to contribute to abnormal development during embryogenesis, more and more evidence showed oxidative stress could be an important pathogenic mechanism of neurological and developmental deficits in both animal and human.

Taken effects on fish antioxidant defense system following common freshwater pesticides (atrazine) exposure for example, usually the detected endpoint contains antioxidant enzymatic activities and genotoxicity, which had been performed in zebrafish *Danio rerio* (Jin et al. 2010), common carp *Cyprinus carpio* (Chen et al. 2015), and so on. Liver was one of the main target organ (Salaberria et al. 2009; Jin et al. 2012; Kroon et al. 2014), but hepatic transcripts was not always affected by androgenic compounds when faced to oxidative stress (Albertsson et al. 2010). Nile tilapia, *Oreochromis niloticus* is sensitive to the oxidative stress of pollutants and can be treated as a kind of ideal material for toxicity experiment (Meng et al. 2014a, b). Our previous study showed that hepatic SOD, CAT and GPx activities and their transcripts were increased in Nile tilapia under methomyl exposure (Meng et al. 2014a, b). GIFT strain of Nile tilapia (“GIFT tilapia” for short), a tropical species, are suitable for culture in warm waters, and very sensitive to aquatic environmental factors (Ma et al. 2015; Gabriel et al. 2015). The normal dose (MT) used in fish farming was 60 mg/L (Rivero-Wendt et al. 2013), and now the residual MT could be detected in waste water obtained from the Beijing area of China (4.1–7.0 ng/L; Sun et al. 2010). A decrease in egg laying rate of female Japanese quails (*Coturnix cotumix japonica*) and the fertility of male Japanese quails when exposed to 50–110 mg/L of MT for 3 weeks (Homklin et al. 2012). The minor deficit of the former studies only used antioxidative enzymatic activities to perform the study (Meng et al. 2014a, b; Ma et al. 2015; Gabriel et al. 2015), and fish genotoxicity may be more sensitive to pollution (Chakravarthy et al. 2014). We know MT has the potential to induce oxidative stress, the main purpose of the present study was to investigate the hepatic genotoxicity (transcriptional) and antioxidant enzymatic signature (post transcriptional) of freshwater GIFT tilapia *O. niloticus* juveniles responding to 0.5, 5 mg/L MT exposure. The present study will also detect other antioxidant parameters to further testify that hepatic antioxidant defense system was impaired following MT exposure.

## Methods

### Experimental design

Fertilized eggs of GIFT tilapia, *O. niloticus* were obtained from Freshwater Fisheries Research Center of the Chinese Academy of Fishery Sciences, Yixing. One-month old *O. niloticus* juveniles were used in the experiment and which were acclimatized in the aquarium facility with dechlorinated tap water at 25 ± 1 °C, with 14 h:10 h light/dark cycle. The experimental fish were offered feed once a day, and the feed purchased from Jiangsu Zhe Ya Food. Co. Ltd, China. Fish (from 4.04 to 4.97 g) were randomly selected for exposure experiments. Throughout the experimental period, water samples were taken before and after each water change, and the experimental conditions were as follows: pH, 7.1 ± 0.5 U; dissolved oxygen (tested by YSI 556MPS, USA), 7.16 ± 0.16 mg/L; total phosphate, 2.16 ± 0.17 mg/L; total nitrogen and ammonia nitrogen (by Nessler’s reagent spectrophotometry), 0.52 ± 0.15 and 0.44 ± 0.06 mg/L respectively; total water hardness (ICP-OES, Optima 7000, PerkinElmer, USA), 194.3 ± 13.0 mg/L CaCO_3_.

The GIFT tilapia juveniles (*n* = 360) were assigned to nine groups (*n* = 40 per aquarium). MT was purchased from Sigma-Aldrich (St Louis, MO, USA). One out of three group fish were exposed to 0.5 mg/L MT and 5 mg/L MT respectively, and the last were reared in water without MT treatment in triplicate. Fish were exposed to test solutions for 21 days and all of the exposure solutions were replaced every 48 h with the fresh exposure solutions of the same concentration during the exposure experiment. The control group was also kept for 21 days with changing fresh water without MT every 48 h. There were no statistically significant differences in bodyweight or length in the exposure experiment. During the experiment, no fish mortality was observed. From the initial exposure day, sampled the water once 2 days both in the control groups and the experimental groups in triplicate.

### Fish sampling

All fish liver samples of the exposure and control groups were collected once a week. In each group per sampling point, fish liver were sampled for gene expression (*n* = 6) and biochemical analysis (*n* = 6) respectively. Particularly samples for gene expression studies were homogenized using Trizol reagent (Invitrogen, USA), frozen in liquid nitrogen and stored at −80 °C immediately until utilization.

### Determination of oxidative stress

For biochemical analyses, liver from 6 individuals per group at every sampling point was washed with ice-cold physiological salt water (0.86 % NaCl) thoroughly, then dried the surface with absorbent paper, weighed and killed unconsciously by a sharp blow on the head. Whole liver samples were homogenized on ice with cold 0.86 % physiological salt water (1:9, w/v), and then centrifuged at 2, 500 r/min at 4 °C for 10 min. The supernatant was analyzed for the activity assays of CAT, GPx, GR, GST, SOD and the content of MDA, GSH, the total protein and the total antioxidant capacity using the commercial kits purchased from Nanjing Jiancheng Bioengineering Institute in triplicate (Nanjing, China). The experiment were quantified spectrophotometrically with a PowerWave XS2 (BioTek instruments Inc, Vermont, USA).

The total protein content (recorded at 595 nm) was determined using Coomassie Brilliant Blue G-250 staining (Bradford 1976). MDA content was measured by assaying the decomposition product of polyunsaturated fatty acid hydroperoxides was determined by the TBA reaction as described by Luo et al. (2006). According to the directions, the mixture was heated at constant temperature at 95 °C for 40 min, cooled by running water and centrifuged at 3500 r/min for 10 min. The absorbance of the supernatant was recorded at 532 nm. GSH (the total glutathione) content was quantified using the method of reacting with 5,5-dithiobis-2-nitrobenzoic acid (DTNB) (Beutler and Kelly 1963) and the generated yellow compound’s absorbance was recorded at 420 nm.

SOD activity was measured through the method of WST-1 by inhibiting of nitroblue tetrazolium reduction at 450 nm (Huang et al. 2007). The final concentration consisted of 50 mM sodium phosphate buffer, 0.1 mM EDTA, 0.01 mM cytochrome c, 0.05 mM xanthine, and 0.005 mM xanthine oxidase. The reaction was initiated when xanthine oxidase was added to the enzyme extract at 25 °C. One unit of SOD activity is defined as the amount of enzyme required to inhibit the oxidation reaction by 50 % and is expressed as U/mg protein. CAT activity was determined by measuring hydrogen peroxide based on the production of its stable complex with ammonium molybdate at 405 nm (Góth 1991). The reaction system consisted of 50 mM sodium phosphate buffer (pH 7.0) and 19 mM hydrogen peroxide. The reaction was quantified at 25 °C by measuring the disappearance of H_2_O_2_. GPx activity was assayed with the spectrophotometer by surveying the decrement of the glutathione’s enzymatic reaction at 412 nm. One unit (U) of CAT and GPx activity is defined as the amount of enzyme consuming 1 μmol of substrate or generating 1 μmol of product per minute and refereed per milligram soluble protein (U/mg protein). GR activity was determined by monitoring the glutathione-dependent oxidation of NADPH at 340 nm (Schaedle 1977). GST activity was measured using 1-chloro-2,4-dinitrobenzene (CDNB) as a substrate (Zhang et al. 2004), and the enzyme activity was determined by monitoring changes in absorbance at 412 nm. The assay contains 100 mM sodium phosphate buffer (pH 6.5), 60 mM glutathione (GSH), and 60 mM CDNB (dissolved in ethanol). One unit of GST activity was calculated as the amount of enzyme catalysing the conjugation of 1 μmol of CDNB with GSH per minute at 25 °C.

### RNA extraction, reverse transcription (RT) and qRT-PCR

Total RNAs were extracted from all fish liver of GIFT tilapia juveniles from MT exposure and the control groups with Trizol reagent (Invitrogen, USA) and further treated with RNase-free DNase I (Fermentas, Canada). To check for genomic DNA contamination and to verify the total RNA quality, we loaded the total RNA in a 1 % agarose gel with EtBr (Sigma-Aldrich, USA) staining and checked the 18/28S ribosomal RNAs integrity together with the normal check using the spectrophotometric method (NanoDrop 1000, Thermo Scientific, USA). After RNA quality was determined, the cDNAs were synthesized from 3 μg DNase I-treated total RNA using the M-MLV First Strand cDNA Kit (Invitrogen, USA) with the oligo(dT)_12–18_ primers in a 20 μL final volume according to the instruction manual. The cDNAs were used for cloning genes and carried out analysis of gene expressions after normalization.

The qRT-PCR was performed by CFX96 Real-Time PCR System (Bio-Rad, USA) with SYBR (TaKaRa, Japan). After the normalization of each cDNA samples, the qRT-PCR reactions were carried out with 1× SYBR Premix Ex Taq™, 0.4 μM of each primer, and 2.5 μL RT reaction solution in a final volume of 25 μL in triplicate. The reaction was initially denatured at 95 °C for 30 s, followed by 40 cycles of denaturation at 95 °C for 5 s and annealing at 60 °C for 30 s. A melt curve analysis was performed at the end of each PCR thermal profile to assess the specificity of amplification.

*β*-*Actin* was the most stable reference gene under exposure of MT in our study with the selecting method described in Zheng et al. (2014) (the detail was not revealed). The qRT-PCR primers for *β*-*actin*, *sod*, *cat*, *gpx1*, *gr* and *gst* are mentioned in Additional file 1: Table S1. Each transcript was analyzed on six individuals per each sampling point per each group. The changes of expression levels of these antioxidant genes after MT exposure were calculated by the 2^−ΔΔCt^ method with the formula, F = 2^−ΔΔCt^, ΔΔCt = (C_t, target gene_ − C_t, β-actin_)_MT_ − (C_t, target gene_ − C_t, β-actin_)_control_ (Livak and Schmittgen 2001).

### Statistical analysis

All the experimental data are shown as the mean ± standard deviation of the mean (SD). Data were tested for normality of distribution (Shapiro–Wilk test) and homogeneity of variance (Levene’s test) prior to analysis. The data were dealt with one-way ANOVA analysis followed by the LSD test (Ahmad et al. 2006) with SPSS Statistics 18.0 (SPSS Inc., Chicago, IL USA), with *P* < 0.05 indicating an significant difference. Data that did not conform to assumptions of normality and homoscedasticity were transformed (lg) and then analyzed as narrated above.

## Results

### Antioxidant parameters following MT exposure

**Fig. 1:**
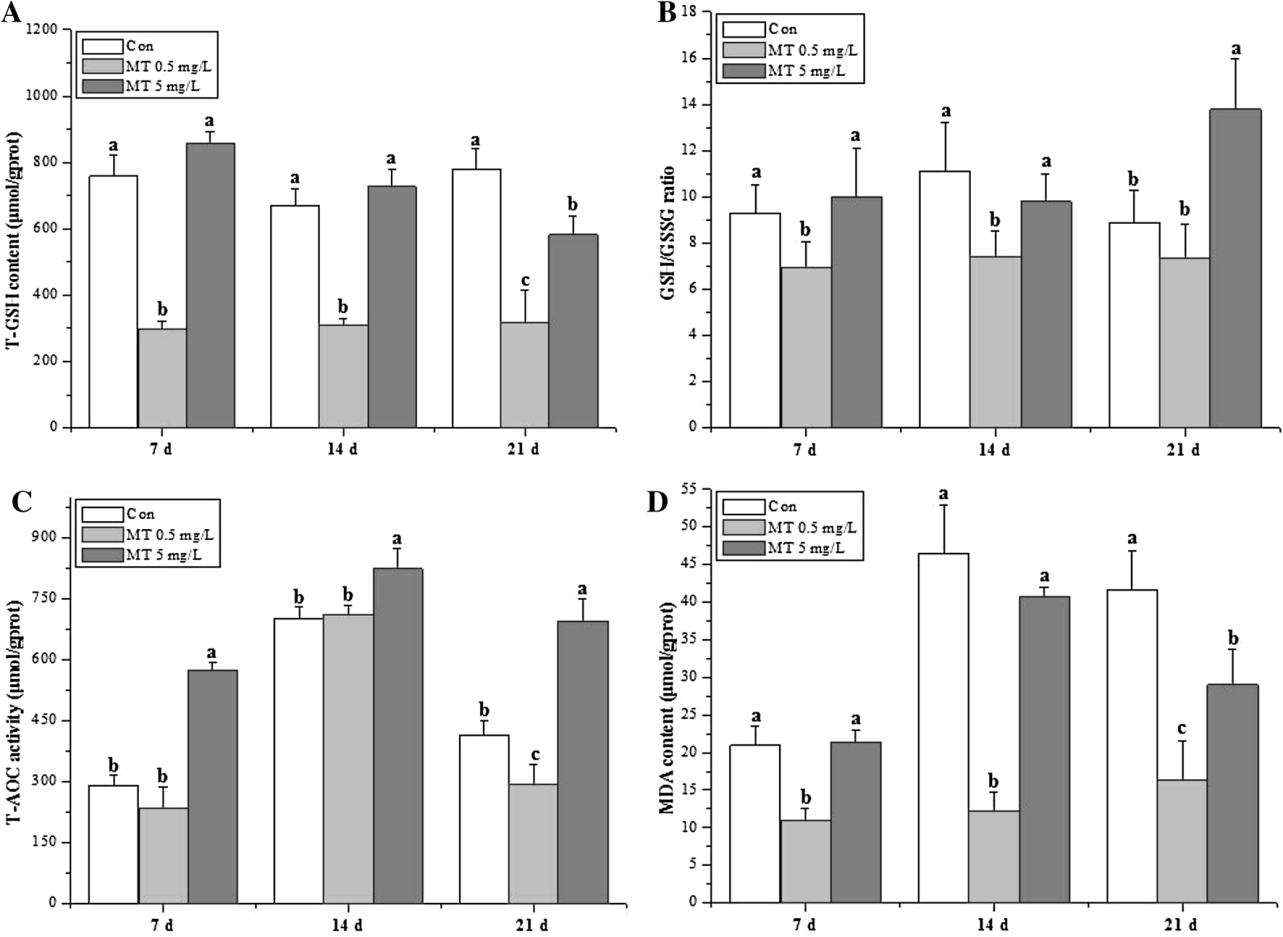
Antioxidative index of *O. niloticus* juveniles under MT exposure. **A** T-GSH, **B** GSH/GSSG, **C** T-AOC, **D** MDA. The *different lower letters* indicate statistically highly significant differences (*P* < 0.05)

The antioxidant parameters (T-GSH, GSH/GSSG, T-AOC and MDA) were showed in Fig. 1. T-GSH, GSH/GSSG and MDA were significant decreased in 0.5 mg/L MT exposure groups at 7 and 14 days. T-GSH and MDA were significant decreased and increased for 0.5 and 5 mg/L MT exposure groups respectively at 21 days (Fig. 1A, D), while GSH/GSSG only showed significant increment in 5 mg/L MT exposure groups (Fig. 1B). T-AOC revealed significant increments in 5 mg/L MT exposure groups all through the exposure time (Fig. 1C).

### Antioxidant enzymes

**Fig. 2:**
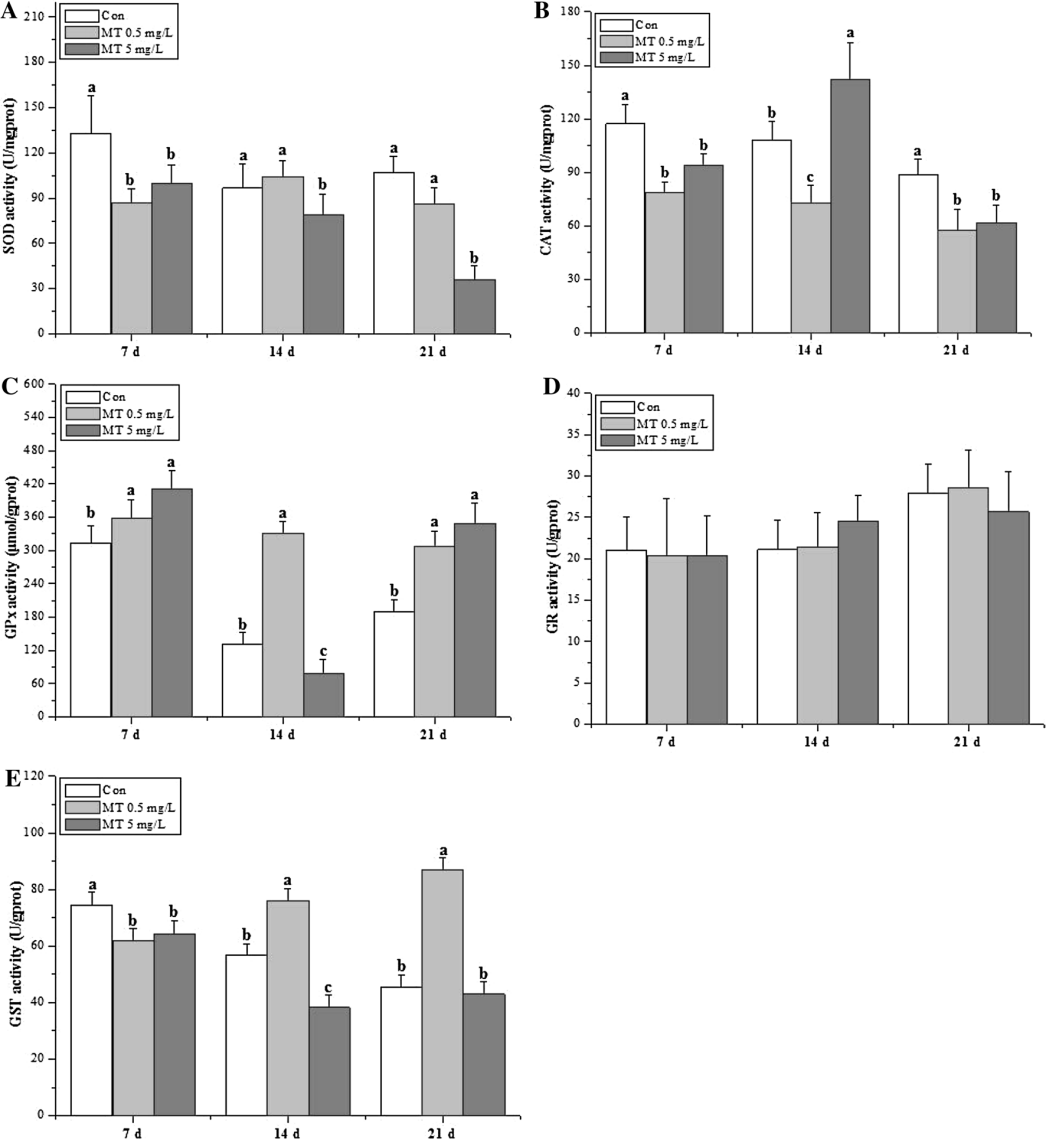
Antioxidative enzymatic activities of *O. niloticus* juveniles under MT exposure. **A** SOD, **B** CAT, **C** GPx, **D** GR, **E** GST

The activities of the antioxidant enzymes (SOD, CAT, GPx, GR and GST) were revealed in Fig. 2. SOD, CAT and GST activities showed significant decreases for MT exposure at 7 days, while GPx activities showed significant increments (by 115 and 131 % for 0.5 and 5 mg/L MT exposure groups respectively, Fig. 2C). GPx activities showed significant increments for MT exposure both at 14 days (0.5 mg/L) and 21 days (0.5 and 5 mg/L), while SOD activities was significant decreased for 5 mg/L MT exposure groups (Fig. 2A). CAT activities demonstrated significant decreases for MT exposure both at 14 days (0.5 mg/L) and 21 days (0.5 and 5 mg/L). CAT activities (increased by 131 %) only showed significant increments for 5 mg/L MT exposure at 14 days (Fig. 2B), while GST activities only showed significant increments for 0.5 mg/L MT exposure at 14 and 21 days (Fig. 2E). GR activities were not affected all through the whole exposure period (Fig. 2D).

### Genotoxicology

**Fig. 3:**
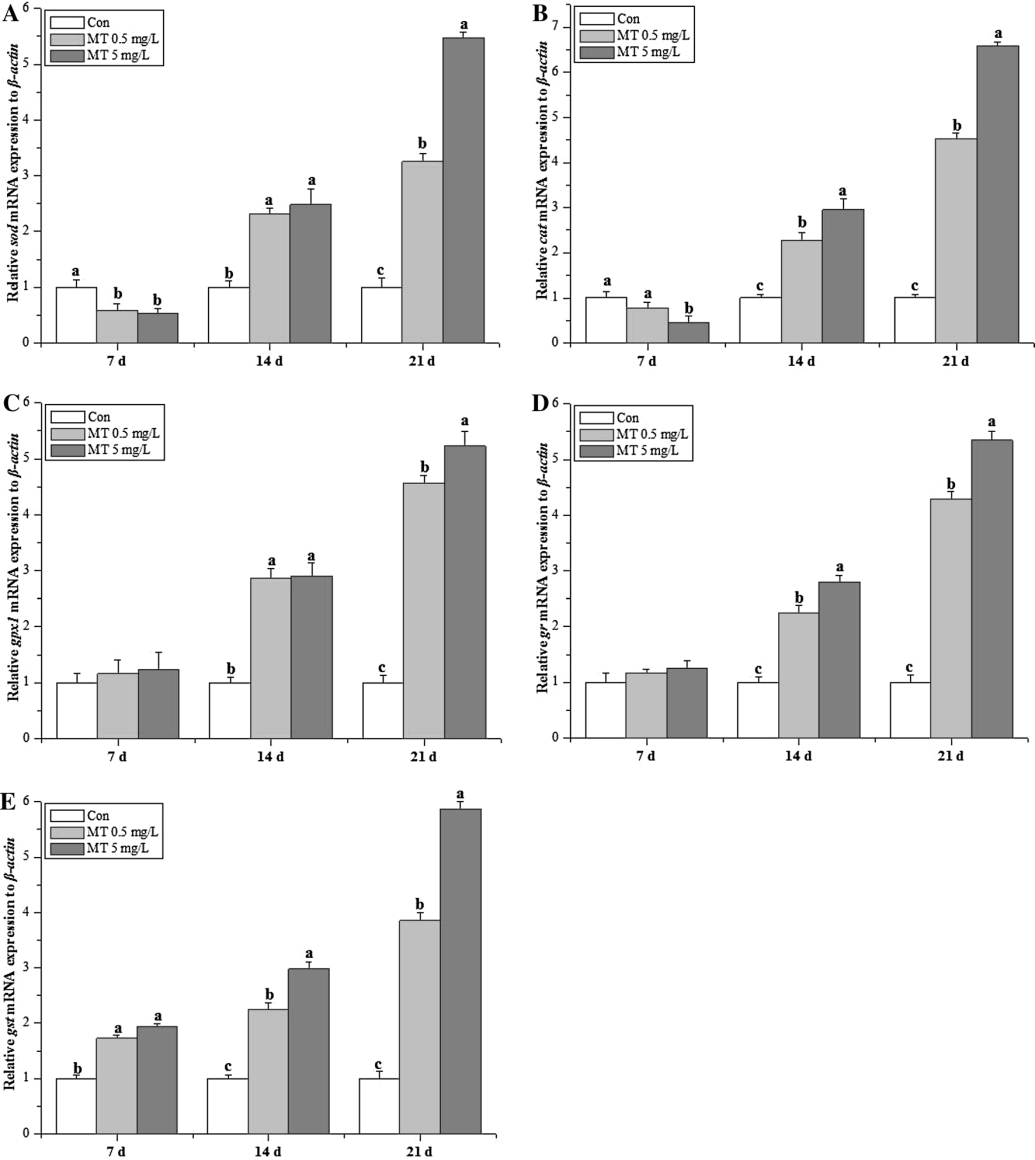
Transcriptional gene expression profiles of *O. niloticus* juveniles under MT exposure. **A** *sod*, **B** *cat*, **C** *gpx1*, **D** *gr*, **E** *gst*

The gene expression profiles of the antioxidant enzymes (*sod*, *cat*, *gr*, *gpx1* and *gst*) were revealed in Fig. 3. *sod* and *cat* showed significant decreases for MT exposure at 7 days (Fig. 3A, B), while *gst* showed significant increments (Fig. 3E). Except for *sod* and *gpx1* transcripts at 14 days (Fig. 3A, C), all of the antioxidant enzymatic genes detected in the present study showed significant increments for MT exposure both at 14 and 21 days, and the genotoxicity profile of antioxidant enzymatic genes revealed dose-dependent manner. *gr* and *gpx1* transcripts were not affected at 7 days (Fig. 3C, D).

## Discussion

SOD and CAT comprise the first-line defense against oxygen toxicity and serve as early indicators of exposure to pollutants that trigger oxidative stress. GSH prevents free radical damage and helps detoxification by conjugating with chemicals, and GST is an important phase II detoxification enzyme in the form of conjugation with glutathione to produce less toxic and more water soluble compounds. Androgenic compounds directly induced antioxidant enzymatic activities and transcriptional genotoxicity (Larsson et al. 2002). Previous studies have used Nile tilapia as chemical test model to further testify the mode of action in the metabolic mechanism of xenobiotic pollutants (Meng et al. 2014a, b). The present study presented hepatic sensitive biomarkers of juvenile GIFT tilapia following MT exposure in the form of antioxidant enzyme activities and transcripts. The antioxidative index (Fig. 1) in 5 mg/L MT exposure groups was significant higher than those in 0.5 mg/L MT exposure groups, while some antioxidative enzymatic activities (SOD, GPx, GST) in 5 mg/L MT exposure groups was significant lower than those in 0.5 mg/L MT exposure groups at 14 days. The genotoxicity data showed that these detected parameters presented the dose-dependent manner at 14 days (except for *sod*, *gpx1*) and 21 days. To conclude, genotoxicity was more sensitive than enzymatic parameters based on the data observed in the current study (Chakravarthy et al. 2014).

SOD, CAT and GST activities showed almost the same tendency in the present study, which provided inconsistent data to the study demonstrated as different response under chlorpyrifos exposure (Jin et al. 2015). CAT and GPx can act cooperatively as scavengers of H_2_O_2_ and other hydroperoxides. Vieira et al. (2012) reported the decreased CAT activity was concomitant with the stimulated SOD and GPx activity in goldfish under acute toxicity of manganese exposure. The present study showed the reverse tendency between SOD, CAT activities with GPx activity for treated groups at 7 days, especially in treated groups at 14 days in the present study, which demonstrated that CAT activity could be compensated by a high decrease of the GPx activity (Atli and Canli 2011). GST activity was also significantly induced at 5 mg/L MT groups, which in agreement with the study observed in *Leuciscus cephalus* exposed to heavy metals (Hermenean et al. 2015). The decreased total GSH observed in the present study was the same as methylmercury-exposed in rainbow trout (Mozhdeganloo et al. 2015), while different from waterborne lead exposure for tilapia (Kaya and Akbulut 2015). The alteration of GSH content and metabolism in different studies suggest that GSH has a key role in oxidative-induced toxicity caused by MT. The reduction in the levels of MDA in the present study is not in agreement with the study performed by Mozhdeganloo et al. (2015). The decrease in the GSH/GSSG ratio for 0.5 mg/L MT exposure groups in the present study, implied the oxidation of GSH to GSSG with the signal of increased detoxification of ROS (Guzmán-Guillén et al. 2013), which was the same as tilapia liver under spinosad exposure (Piner and Üner 2013).

The transcriptional changes in these genes in the liver could be good biomarkers for stress levels in *O. javanicus* exposed to iprobenfos (Woo et al. 2009), and this study indicates that transcripts of the detected antioxidant enzymes were significant increased except for *sod* and *cat*. The down-regulation of *sod* and *cat* was the same as the study performed in juvenile Jian carp challenging against dietary choline (Wu et al. 2014). However, the up-regulate *sod*, *cat* and *gr* mRNA levels, which suggesting an adaptive mechanism against stress in Jian carp (Jiang et al. 2014). The transcriptional and functional responses of antioxidant enzymes are inversely correlated in GIFT tilapia when exposed to MT, which was demonstrated in common carp exposed to organochlorine pesticides (Karaca et al. 2014). The dose-dependent pattern revealed in the present study was the same as the study of benzo[a]pyrene on marine medaka *Oryzias melastigma* (Kim et al. 2014), atrazine exposure on zebrafish (Jin et al. 2010), methomyl on Nile tilapia (Meng et al. 2014a, b). This study indicates that although MT stimulates adaptive increases in the expression of some antioxidant enzyme genes, it also induces oxidation and the depletion of most activities of antioxidant enzyme (Fig. 2a, b except for 5 mg/L MT at 14 days, Fig. 2e except for 0.5 mg/L MT at 14, 21 days) and GSH content (Fig. 1a) due to the increase of ROS production (Meng et al. 2014a, b; Mukhopadhyay et al. 2015). To conclude, genotoxicity are reliable environmental biomarkers for MT induced oxidative stress in tilapia juveniles, while SOD, CAT, GPx, GST, T-AOC (5 mg/L MT) also be sensitive for MT exposure. Therefore, the useful biological indicators of environmental MT contamination revealed for the aquatic ecosystem (Kayode et al. 2014).

## Conclusion

Antioxidative parameters (T-GSH, GSH/GSSG and MDA) and antioxidant enzymes (SOD, CAT and GST) significantly decreased under MT exposure, and the genotoxicity profile of antioxidant enzymatic genes revealed as the dose-dependent manner. The current study presented evidence that MT could result in oxidative stress response in the early stages of GIFT tilapia.

## Additional file

10.1186/s40064-016-1946-6 The primers of tilapia used for qRT-PCR in the study.
