# Supplementary figures and images for: Effect of 17α-methyltestosterone (MT) on oxidation stress in the liver of juvenile GIFT tilapia, Oreochromis niloticus
Source: Springerplus. 2016 Mar 15;5:338. doi: 10.1186/s40064-016-1946-6 (PMC4792819; doi:10.1186/s40064-016-1946-6)

**Supplementary table legend**

Table S1. The primers of tilapia used for qRT-PCR in the study


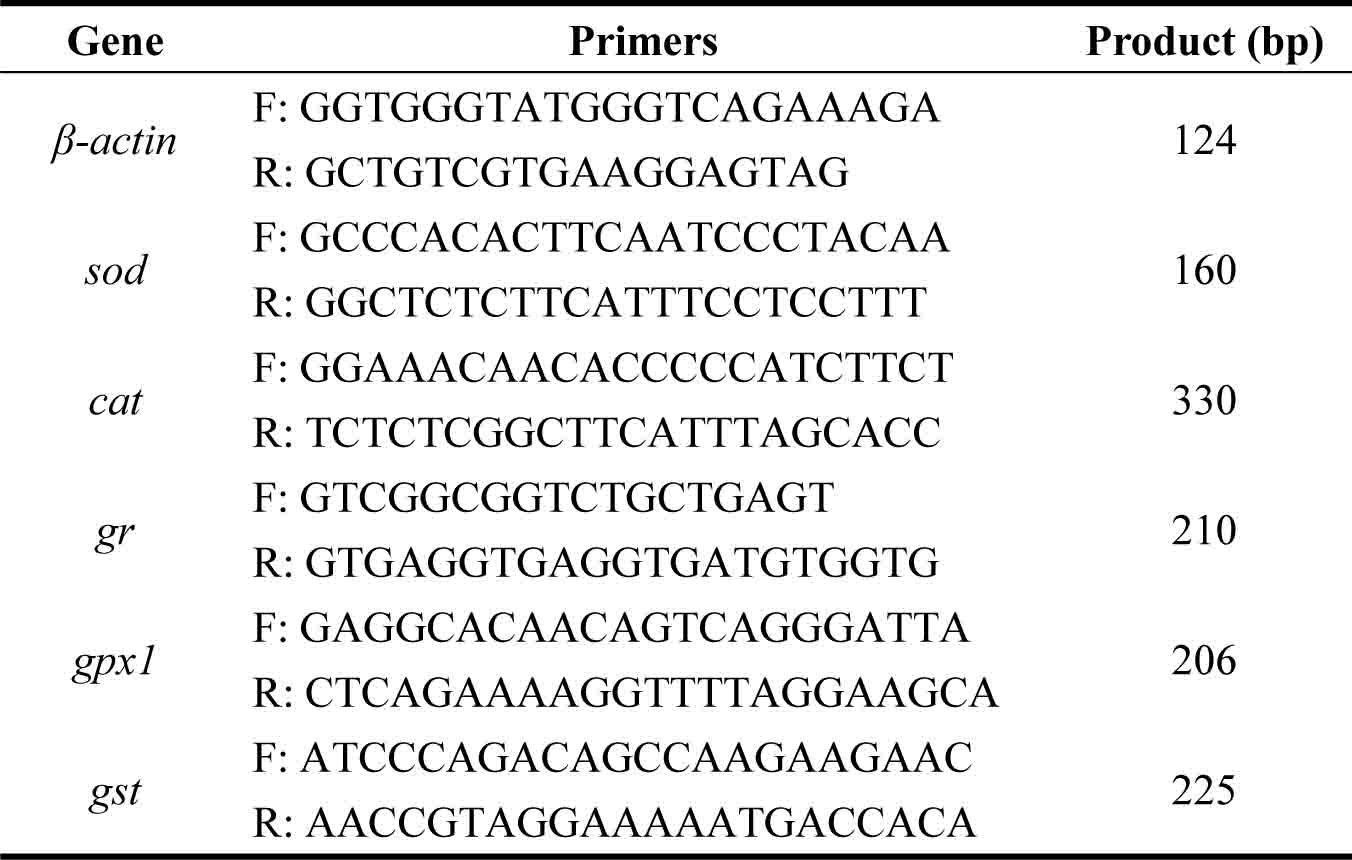

Supplement: Supplementary file 1 — 10.1186/s40064-016-1946-6 The primers of tilapia used for qRT-PCR in the study. [file 40064_2016_1946_MOESM1_ESM.doc]
